# Topological acoustic synapse for high-dimensional neuromorphic computing

**DOI:** 10.1126/sciadv.aec6633

**Published:** 2026-06-12

**Authors:** Jinli Chen, Akinsanmi S. Ige, Keith Runge, Pierre A. Deymier, Xiaodong Yan

**Affiliations:** ^1^ Department of Materials Science and Engineering, University of Arizona, Tucson, AZ 85721, USA.; ^2^ New Frontiers of Sound Science and Technology Center, University of Arizona, Tucson, AZ 85721, USA.; ^3^ Department of Electrical and Computer Engineering, University of Arizona, Tucson, AZ 85721, USA.; ^4^ James C. Wyant College of Optical Sciences, University of Arizona, Tucson, AZ 85721, USA.

## Abstract

The human brain performs complex, high-dimensional (HD) computations, such as causal reasoning, counterfactual thinking, and abstraction, with ~10^11^ neurons while consuming ~20 watts of power. Neuromorphic computing seeks similar efficiency, but current devices face bottlenecks in bandwidth, energy, wiring, footprint, and reliability that limit scalability. Here, we introduce the topological acoustic synapse (TAS), an acoustic-wave neuromorphic device that circumvents these limits by mapping information in multivariate state spaces. A single TAS generates and manipulates numerous computing channels that operate independently and in parallel. The TAS leverages nonlinear interactions to emulate biorealistic neuromorphic functionalities, including reconfigurable synaptic plasticity, neuromodulation, and hybrid analog-digital control. In classification tasks, a TAS handles multiple inputs simultaneously and generates various outputs, converging 20% faster while using 60% fewer parameters and at least an order of magnitude less power than state-of-the-art electrical devices. This work establishes the first acoustic synapse with parallel HD computing capabilities, presenting a scalable paradigm for neuromorphic hardware with high computational density.

## Teaser

Topological acoustic synapses pack many computing channels into one device, enabling parallel, low-power learning.

## INTRODUCTION

The escalating computational demands of artificial intelligence (AI) are driving the search for computing schemes that overcome the von Neumann bottleneck (*1*, *2*), where the physical separation of the processing unit and memory leads to substantial energy consumption and data-transfer latency (*3*). Neuromorphic computing offers a compelling alternative by emulating the brain’s architecture where memory and processing are colocated (*4*, *5*). However, replicating the brain’s sheer scale and parallelism remains the fundamental bottleneck in neuromorphic hardware (*6*, *7*). In the human brain, there are about 10^11^ neurons (*8*), with hundreds of millions (*9*) operating concurrently to support the HD computing tasks like causal reasoning (*10*), counterfactual thinking (*11*), and abstract thought (*12*). Current neuromorphic devices, like memristors (*13*, *14*), transistors (*15*, *16*), and spintronic oscillators (*17*–*19*), are fundamentally constrained (*7*, *20*). Memristors often suffer from the stochastic switching nature in conductive filament formation and rupture, limited switching endurance, and high device-to-device variations (*21*, *22*). Transistor-based synapses are stable but require complicated circuits, leading to a large physical footprint and notable power consumption (*23*). Emerging neuromorphic devices like spintronic oscillators are constrained by poor network synchronization and vulnerability to noise and cross-talk (*24*–*26*). In addition, these approaches share a critical limitation: Each device typically functions as only a single synapse (*27*) with simple plasticity. These limitations prevent current neuromorphic devices from scaling to the brain’s parallelism and connectivity due to massive energy, footprint, and routing challenges (*7*, *28*–*30*). Therefore, a new physical mechanism that can intrinsically process high-dimensional data within a single device is urgently needed.

Recently, topological acoustic (TA) waves in coupled waveguides have emerged as a physical platform capable of processing large amounts of data (*31*, *32*). The complex interplay among these TA waves creates a rich set of nonlinear modes that function as phase bits (phi-bits). The phi-bits enable numerous computing operations (*33*, *34*), and some of these are analogous to operations in quantum computing (*35*–*37*). Unlike conventional electronic bits, which are discrete and binary (0 or 1) and require a separate physical component for each bit (*38*), phi-bits arise from the collective interactions of waves within a single device (*39*). This allows a single TA device to intrinsically host a multivariate state space, where numerous phi-bits act as independent, parallel channels for computation (*40*). As a result, the TA system offers an ideal platform for HD parallel computing and serves as a natural substrate for physical neural networks (*41*).

Here, we report the experimental realization of a TA synapse (TAS), a TA wave-based device that presents three main advantages over existing neuromorphic hardware. First, by using nonlinear TA wave coupling, a TAS can map multiple inputs to a high-dimensional multivariate state and process them in parallel with the self-generated, multiple independent computing channels. In contrast, a memristor or transistor has only one computing channel (the channel current), providing limited bandwidth. Second, each phi-bit in TAS supports both reconfigurable analog and digital-like operations. This capability enables TAS to emulate biorealistic behaviors, including synaptic plasticity (*42*) with variable learning rates, bilingual synaptic response (*43*), and neuromodulation (*44*) with both fine and coarse control. Third, inherent parallelism and biorealistic functionality allow a TAS to mitigate the area and energy overhead of conventional hardware, demonstrating high accuracy, rapid convergence, and robustness in recognition tasks at remarkably low power. These distinctive features imply that TAS has clear advantages for realizing the dense, scalable, and computationally powerful hardware required for the next generation AI.

## RESULTS

### TAS and phi-bit

**Fig. 1.**
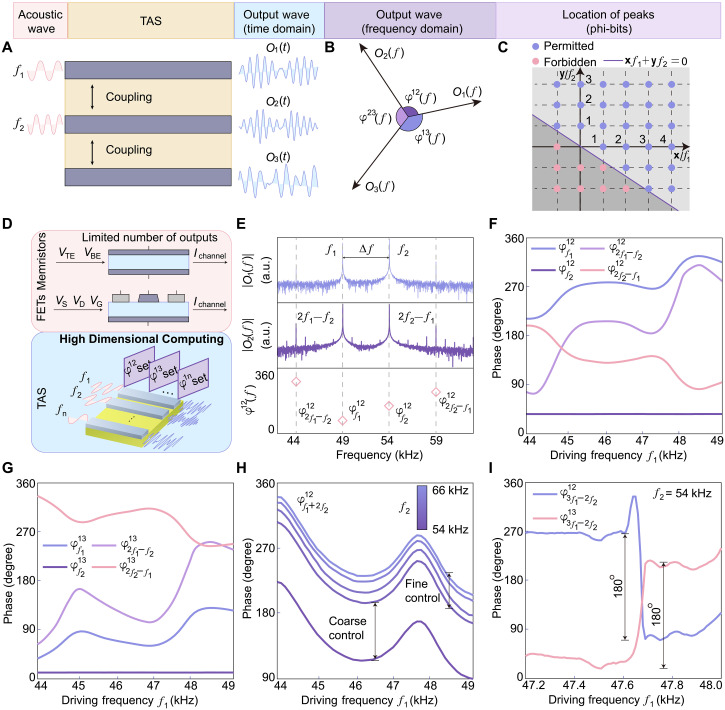
TAS and phi-bits: Definition and manipulation. (**A**) Schematic of the TAS composed of three coupled waveguides. Two input acoustic waves with distinct frequencies (*f*_1_ and *f*_2_) interact nonlinearly to produce three time-domain output signals [*O_k_*(*t*)]. (**B**) In the frequency domain, the output signals are represented as complex phasors [*O_k_*(*f*)], and phi-bit information is mapped in the relative phases [φ*^ij^*(*f*)] between the phasors [*O_i_*(*f*) and *O_j_*(*f*)]. (**C**) The two-dimensional map of phi-bits, showing permitted (blue) and forbidden (pink) frequencies versus the driving frequencies (*f*_1_ and *f*_2_). Permitted frequencies are integer combinations *pf*_1_ *+ qf*_2_, while negative combinations (*pf*_1_ *+ qf*_2_ < 0) are forbidden. Each permitted frequency yields several phi-bits, one per relative phase φ*^ij^*. (**D**) Comparison of TAS and conventional neuromorphic devices. Conventional devices such as FETs and memristors typically have a limited number of outputs (channel current). In contrast, the TAS hosts a HD multivariate computation space composed of numerous independent phi-bit sets. (**E**) Output amplitude spectra and the phi-bits. The top two panels show the spectra for the two output signals, ∣*O*_1_(*f*)∣ and ∣*O*_2_(*f*)∣, with prominent peaks at the frequencies *f*_1_, *f*_2_, 2*f*_1_ − *f*_2_, and 2*f*_2_ − *f*_1_. The bottom panel shows the phi-bit phases extracted at these four peak frequencies. (**F** and **G**) Continuous, nonlinear tuning. With *f*_1_ swept from 44 to 49 kHz, phi-bits in the (F) φ^12^ set and (G) φ^13^ set change smoothly. Specific phi-bit pairs exhibit inversion or linear correlations. (**H**) Multiparameter tuning. The second driving frequency *f*_2_ acts as a control knob for $\varphi_{f1+2f2}^{12}$. The changing rate of $\varphi_{f1+2f2}^{12}$ with respect to *f*_2_ varies between a “coarse control” regime (~60°/kHz) and a “fine control” regime (~1°/kHz). (**I**) Digital-like switching. At a critical frequency of *f*_1_ ≈ 47.6 kHz, the $\varphi_{3f1-2f2}^{12}$ and $\varphi_{3f1-2f2}^{13}$ undergo an abrupt 180° phase shift, demonstrating a digital-like switching.

The TAS consists of three elastically coupled, finite-length acoustic waveguides. As shown in Fig. 1A, injecting two continuous acoustic waves with distinct driving frequencies (*f*_1_ and *f*_2_) results in nonlinear interactions that generate three output signals *O_k_*(*t*) in the time domain (fig. S1). In the frequency domain, these output signals correspond to complex phasors *O_k_*(*f*) (Fig. 1B). Phi-bit information is mapped in the relative phases φ*^ij^*(*f*) between these phasors (Fig. 1B, *ij* is relative index). Phi-bits ($\varphi_{\mathrm{pf}1+q\;f2}^{\mathrm{ij}}$) at specific frequencies *pf*_1_ *+ qf*_2_ (Fig. 1C, *p* and *q* are integers) are ideal computational units due to their exceptional stability and controllability (section S1). Phi-bits with the same *ij* are grouped into a phi-bit set (Fig. 1D, φ^12^set, φ^13^set, and φ^23^set). Each set contains a large number of phi-bits (Fig. 1C) and is independent of other sets. Together, a single TAS intrinsically hosts a multivariate computation space composed of numerous independent computation units, phi-bits, having a distinctive feature compared to traditional electronic devices, which typically have only one computation unit in a device (Fig. 1D, *I*_channel_). This massive, in situ parallelism is what fundamentally separates TAS from traditional devices, such as field-effect transistors (FETs) and memristors.

Figure 1E shows four typical phi-bits in a TAS ($\varphi_{2f1-f2}^{12}$ through $\varphi_{2f2-f1}^{12}$), which are located at prominent peaks (*f*_1_, *f*_2_, 2*f*_1_ − *f*_2_, and 2*f*_2_ − *f*_1_) of output spectra ∣*O*_1_(*f* )∣ and ∣*O*_2_(*f* )∣. The values of the phi-bits are extracted by relative phase between *O*_1_(*f*) and *O*_2_(*f*) (section S1). We begin with a characterization of the TAS’ tunability by sweeping a single driving frequency *f*_1_. Figure 1F shows that $\varphi_{f1}^{12}$, $\varphi_{2f2-f1}^{12}$, and $\varphi_{2f1-f2}^{12}$ evolve in a continuous and nonlinear manner with *f*_1_, whereas the $\varphi_{f2}^{12}$ remains constant. Some phi-bits are independent ($\varphi_{f1}^{12}$ and $\varphi_{f2}^{12}$), while others are correlated in different ways, including an inversion relation between $\varphi_{f1}^{12}$ and $\varphi_{2f2-f1}^{12}$ and a linear correlation between $\varphi_{f1}^{12}$ and $\varphi_{2f1-f2}^{12}$. The phi-bits in the φ^13^ set exhibit similar tunability and correlations, and φ^13^ set evolves independently of the φ^12^ set (Fig. 1G). The second driving frequency (*f*_2_) provides another degree of freedom in modulating TAS. Figure 1H shows that when *f*_2_ varies around 54 kHz, $\varphi_{f1+2f2}^{12}$ enters a “fine control” regime with a changing rate less than 1°/kHz, when *f*_2_ varies around 66 kHz, $\varphi_{f1+2f2}^{12}$ enters a “coarse control” regime with a changing rate of ~60°/kHz. Beyond these continuous controls, TAS exhibits digital-like switching capabilities. As shown in Fig. 1I, at *f*_1_ = 47.6 kHz, $\varphi_{3f1-2f2}^{12}$ and $\varphi_{3f1-2f2}^{13}$ undergo an abrupt phase shift of π radians (180°). This binary, switch-like behavior provides a straightforward mechanism for TAS to implement Fourier transformations (*45*) and logic operations like permutation gates (*40*). The analog and digital behaviors of phi-bits within a TAS make it suitable for emulating biological synapses and implementing neuromorphic applications.

### TAS for two terminal synapse and parallel processing

**Fig. 2.**
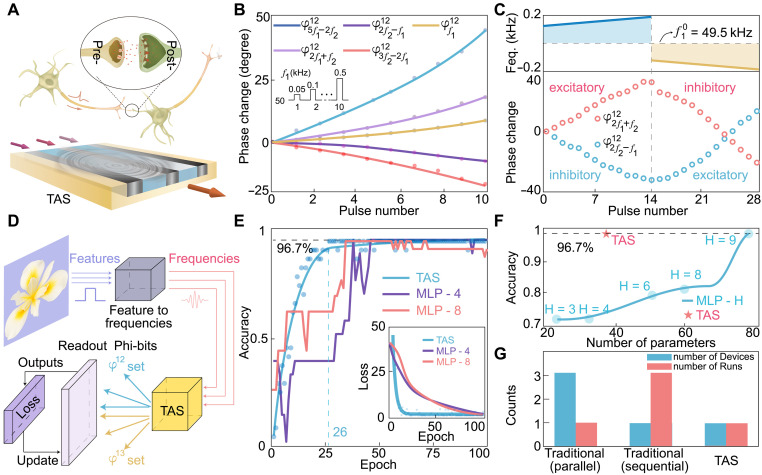
Neuromorphic functionality and computational benchmarking of TAS. (**A**) Schematic comparing the TAS with a biological synapse. A continuous acoustic input whose driving frequency is pulse-modulated acts as the presynaptic signal, and the resulting phi-bit phase state represents the tunable synaptic weight. (**B**) Emulation of multilevel synaptic plasticity. A series of 1-s input pulses (inset) induces a range of progressive changes in phi-bit phases, analogous to long-term potentiation (LTP) and long-term depression (LTD). Different phi-bits in TAS exhibit distinct plasticity, from strong potentiation (blue) to varying degrees of depression (red, purple). (**C**) Reconfigurable synaptic response. The *f*_1_ is modulated in a pulse-like manner, ramping up over pulses 1 to 14 and down over 15 to 28 (top panel). During pulses 1 to 14, $\varphi_{2f1+f2}^{12}$ is excitatory and $\varphi_{2f2-f1}^{12}$ is inhibitory. During pulses 15 to 28 these roles invert, evidencing controllable excitation-inhibition switching. (**D**) Schematic of the workflow for the Iris classification task. Distinct data features are mapped into separate driving frequencies. The resulting HD phi-bits are then processed by a linear readout layer that performs the final classification. (**E**) Classification accuracy and loss (inset) versus training epoch for the TAS model and two MLP benchmarks (MLP-4 and MLP-8). The TAS converges faster, reaching 96.7% accuracy in 26 epochs. (**F**) Parameter efficiency. MLPs with different hidden-layer sizes (MLP-H) are benchmarked as baselines and show increasing accuracy with more parameters. TAS reaches 96.7% accuracy with 39 parameters, whereas matching this performance requires an MLP-9 with 75 parameters. (**G**) Comparison of hardware resources required to process parallel data streams. TAS’s inherent parallelism allows it to complete the task with a single device in a single run. This is achieved when the number of waveguides matches the number of required features, in contrast to traditional parallel (multiple devices) or sequential (multiple runs) approaches.

TAS uses nonlinear TA wave interaction to modulate the phi-bit phase, mimicking synaptic potentiation and depression (*46*) (Fig. 2A). In neuroscience, potentiation and depression refer to activity-induced changes in synaptic strength. Potentiation means the synapse becomes stronger and produces a larger response to the input, while depression means the synapse becomes weaker and produces a smaller response (*47*). Potentiation and depression provide a way to emphasize informative signals and suppress less informative ones in a computation. Figure 2B demonstrates how the TAS emulates finely tuned, multilevel synaptic plasticity. A continuous input acoustic wave is applied to TAS, and its driving frequency *f*_1_ is modulated in a pulse-like manner (fig. S2). Each pulse has a duration of 1 s and a period of 3 s. The baseline of *f*_1_ is 50 kHz. For the first pulse, *f*_1_ is incremented by 0.05 kHz, and this incremental step increases by an additional 0.05 kHz for each subsequent pulse. Specifically, the TAS exhibits highly reconfigurable long-term potentiation (LTP) and long-term depression (LTD) (*43*, *46*), which describe potentiation and depression that persist after repeated stimulation. The device’s response can be tuned from strong potentiation (blue line) to moderate (purple line) and weak potentiation (yellow line). Furthermore, it can be configured to produce a range of depressive states, from strong depression (red line) to moderate depression (dark blue line). Figure 2C demonstrates that the TAS can realize both a bilingual synaptic response (*43*) and dynamic, input-dependent switching between excitatory and inhibitory responses. Excitatory and inhibitory responses describe whether an input increases or decreases the output, and switching between them enables controlled amplification and suppression across channels (*48*). A continuous acoustic wave with the driving frequency modulation following a 28-pulse-train shape is applied to TAS (Fig. 2C, top panel), with the frequency increasing monotonically for the first 14 pulses and then decreasing for the final 14 pulses. During pulses 1 to 14, $\varphi_{2f1+f2}^{12}$ is in excitatory mode while $\varphi_{2f2-f1}^{12}$ is in inhibitory mode. During pulses 15 to 28, $\varphi_{2f1+f2}^{12}$ switches to inhibitory mode and $\varphi_{2f2-f1}^{12}$ switches to excitatory mode. This reconfigurable, bidirectional behavior highlights TAS’s controllable excitation-inhibition switching.

To demonstrate how the TAS’s synaptic behavior enables parallel computing, we implemented a hybrid framework for an iris classification task (*49*), as illustrated in Fig. 2D. Distinct features extracted from the input data are mapped onto separate driving frequencies. TAS processes all the input frequencies simultaneously and generates independent φ^12^ set and φ^13^ set that form a high-dimensional representation of the input features. The resulting parallel outputs are then interpreted by a trainable linear readout layer composed of three neurons to perform the final classification. Only the linear readout layer is trained here, whereas the TAS operates as a fixed physical feature expander (table S1). For comparison, the same task is performed with conventional multilayer perceptron (MLP) of different sizes. In these MLP baselines, all network weights, including those in the hidden layers and the output layer, are optimized during training. The MLPs therefore have substantially more trainable parameters than the single linear readout used with TAS. The TAS outperforms MLP in iris classification, achieving a 96.7% classification accuracy in only 26 training epochs (Fig. 2E). Two MLPs, with hidden layers of four and eight neurons, respectively (MLP-4 and MLP-8), are benchmarked. The TAS converges 20% faster and more steadily than MLPs. This advanced performance stems from the high-dimensional computing channels that are uniquely generated by TAS, where the TAS transforms the inputs into a multivariate space where the classification becomes more linearly separable, allowing a simpler readout layer to converge on the solution with improved speed and stability.

TAS demonstrates superior efficiency in both trainable parameters and physical resources. Figure 2F highlights the efficiency in parameters. The TAS achieves a final accuracy of 96.7% using only 39 trainable parameters. In contrast, MLP-8 uses 67 parameters yet fails to achieve this performance. To achieve comparable accuracy, an MLP would require an even larger nine-neuron hidden layer (an MLP-9 architecture) and notably more parameters. Figure 2G illustrates the hardware resource efficiency by comparing the resources required to process three parallel data streams using three different schemes. A traditional parallel approach requires multiple physical devices working simultaneously, a method that is fast but requires more hardware. A traditional sequential approach uses a single device repeatedly, which saves hardware but requires a considerable time cost from multiple runs. TAS achieves hardware parallelism, performing the same task with only one device and in one run. This efficiency highlights the TAS’s advantage in processing data simultaneously, positioning it as a promising candidate for big-data tasks.

### TAS for neuromodulated synapse

**Fig. 3.**
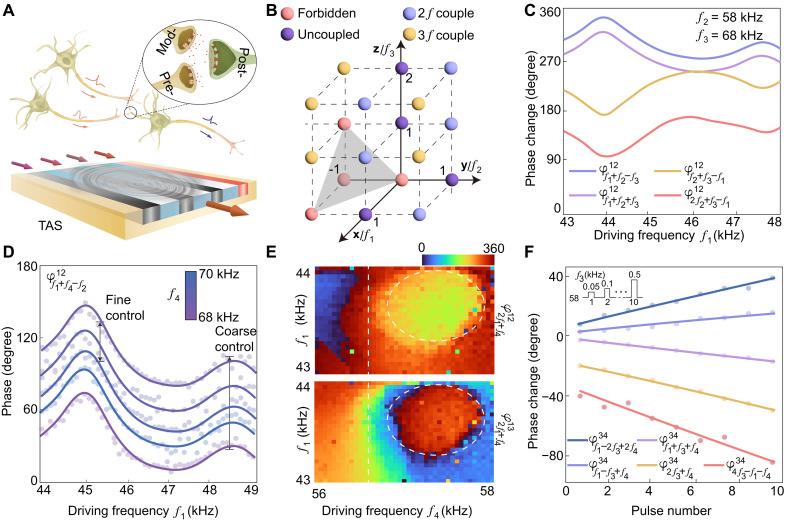
High-dimensionality and neuromodulation in four-waveguide TAS. (**A**) Schematic comparing a four-waveguide TAS to a biological synapse with a modulatory neuron (“Mod-”). The additional acoustic input provides a physical analog for neuromodulation, allowing a third signal to dynamically reconfigure the synaptic response. (**B**) The phi-bit lattice for TAS. With three distinct driving frequencies (*f*_1_, *f*_2_, and *f*_3_), accessible phi-bits occur at integer combination frequencies *pf*_1_ *+ qf*_2_ *+ rf*_3_. The lattice consists of uncoupled (purple), two-frequency coupled (blue), and higher-order three-frequency coupled (yellow) states, which correspond to different orders of nonlinear wave interaction. The three-frequency coupled states, which were not accessible with the two-drive configuration, provide higher-order control of phi-bit behavior. (**C**) Continuous analog tuning of three-frequency coupled phi-bits. The plot shows the phase of several three-frequency coupled phi-bits versus the driving frequency *f*_1_ from 43 to 48 kHz, with *f*_2_ (58 kHz) and *f*_3_ (68 kHz) held constant, demonstrating a smooth, nonlinear response. $\varphi_{f1+f2+f3}^{12}$ and $\varphi_{f2+f3-f1}^{12}$ demonstrate a crossing that is symmetric about the crossing point, which enables permute gate operation (*53*). (**D**) Neuromodulation in the four-waveguide TAS. A fourth frequency (*f*_4_) acts as a modulatory input, tuning the response of the phi-bit $\varphi_{f1+f4-f2}^{12}$ to a sweep in *f*_1_. The changing rate of $\varphi_{f1+f4-f2}^{12}$ with respect to *f*_4_ varies between a coarse control regime (~140°/kHz) and a fine control regime (~30°/kHz). (**E**) Two-dimensional phase maps of the phi-bits $\varphi_{2f1+f4}^{12}$and $\varphi_{2f1+f4}^{13}$ as a function of the driving frequencies *f*_1_ and *f*_4_. The maps reveal distinct operational regimes, including a region of low sensitivity (dashed circle) and a region of medium and high sensitivity (dashed line). (**F**) Emulation of multilevel synaptic plasticity in the four-waveguide TAS. A series of 1-s *f*_3_ pulses (inset) induces a wide spectrum of plastic responses in the φ^34^ set, from strong potentiation (blue) to strong depression (red).

Neuromodulators are signaling molecules, such as biogenic amines and neuropeptides, that modify the behavior of neural networks (*50*). By binding to specific receptors, neuromodulators can alter the strength and dynamics of synaptic connections, which is crucial for higher-order functions like learning and forming memories. In biological systems, a single synapse can be simultaneously influenced by as many as ten neuromodulators (*51*). This type of synapse determines many high-level brain functions such as context-dependent learning and behavioral state-switching (*52*). Mimicking a synapse influenced by multiple neuromodulators is a challenge for traditional neuromorphic devices, as this would require an impractical number of gate terminals to modulate a single synaptic channel, severely complicating device design and fabrication (*16*). In contrast, TAS can scale up easily to emulate multiple neuromodulators by simply incorporating additional waveguides. As proof of concept, we demonstrate this principle by scaling TAS from three waveguides to four, where the additional waveguide serves as a modulatory input to dynamically control the TAS’s behavior (Fig. 3A).

Adding a waveguide expands the TAS’s computational capacity (Fig. 3B). With three distinct input frequencies (*f*_1_, *f*_2_, and *f*_3_), the accessible phi-bits span a three-dimensional lattice. Some phi-bits are generated by a single frequency, while others are generated by the nonlinear coupling of two or three frequencies. The four-waveguide TAS offers more computation units with more precise controllability compared to the three-waveguide TAS. This allows the TAS to mimic the biological synapses that are controlled by multiple neuromodulators. Figure 3C shows that three-frequency coupled phi-bits exhibit a nonlinear, related tunability with the driving frequency *f*_1_. With more dedicated control, $\varphi_{f1+f2+f3}^{12}$ and $\varphi_{f2+f3-f1}^{12}$ demonstrate a crossing that is symmetric about the crossing point, which enables permute gate operation (*53*). Figure 3D shows the neuromodulator effect (*f*_4_) of phi-bit $\varphi_{f1+f4-f2}^{12}$ in TAS. When *f*_4_ varies around 68 kHz, $\varphi_{f1+f4-f2}^{12}$ enters a coarse control regime with a high sensitivity of ~140°/kHz, and when *f*_4_ varies around 70 kHz, $\varphi_{f1+f4-f2}^{12}$ enters a fine control regime with a reduced sensitivity of ~10°/kHz, offering a much larger tunable range compared to the three-waveguide TAS (Fig. 1H).

We further characterize this modulation by simultaneously sweeping *f*_1_ and *f*_4_ (Fig. 3E). The heatmaps of $\varphi_{2f1+f4}^{12}$ and $\varphi_{2f1+f4}^{13}$ reveal their distinct dynamics. Inside the dashed circles, both $\varphi_{2f1+f4}^{12}$ and $\varphi_{2f1+f4}^{13}$ enter a low-sensitivity regime, in which both phi-bits are stable against the variation in driving frequencies. Along the vertical dashed line, $\varphi_{2f1+f4}^{12}$ enters a medium sensitivity regime, while $\varphi_{2f1+f4}^{13}$ enters a high-sensitivity regime. This versatile dynamic allows TAS to personalize different phi-bits for different computing tasks. From a neuromorphic computing perspective, the TAS can assign different phi-bits to emulate a wide spectrum of neuromodulatory effects, ranging from rapid responses, such as dopamine’s modulation of synaptic strength during reward-based learning (*54*), to slow, long-term responses, such as the structural remodeling of neurons induced by chronic stress hormones (*55*). The four-waveguide TAS demonstrates multilevel synaptic plasticity, as shown in Fig. 3F and fig. S3. The TAS exhibits reconfigurability over the degree of potentiation and depression, ranging from strong potentiation ($\varphi_{f1-2f3+2f4}^{34}$, blue) to strong depression ($\varphi_{4f3-f1-f4}^{34}$, red). The synaptic behavior shows improved linearity compared to the three-waveguide TAS (Fig. 2B), which improves the predictability and stability of the learning algorithm and the accuracy of the final model.

### TAS realizes HD computing and type-specific encoding

HD computing encodes information as large, distributed patterns rather than as single numbers. This makes the encoded information robust to noise and error, resembling associative memory in the brain (*56*). On conventional digital hardware, this approach demands heavy memory movement and numerous computational operations (*57*). TAS performs HD computing with native efficiency, as its parallel phi-bits form a high-dimensional physical vector that can be manipulated through either analog control or digital-like switching. This physical implementation bypasses the bottlenecks of conventional hardware, offering a path toward faster and more energy-efficient HD computing.

**Fig. 4.**
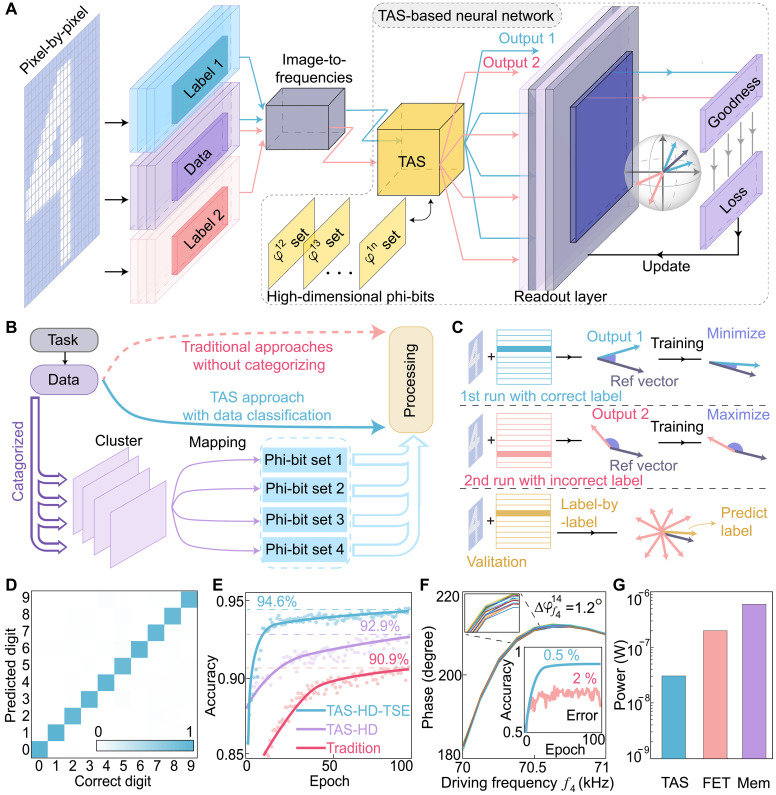
TAS realizes HD computing and TSE. (**A**) Schematic of the physical local learning workflow for the MNIST recognition task. The process involves two parallel runs: One where the input data are paired with its correct label (blue, positive sample) and another where it is paired with an incorrect label (red, negative sample). The data-label pairs are mapped into driving frequencies, which generates HD phi-bit representations. A readout layer processes these outputs to calculate the goodness (cosine similarity), and the difference between the positive and negative scores is used to generate a loss signal. (**B**) Traditional approaches (top, dashed line) typically process uncategorized data uniformly. The TAS approach (bottom, solid line) first categorizes the data into distinct clusters and then maps these clusters to different physical inputs. (**C**) During training, the output vector from a positive sample is trained to minimize its angle to a reference vector, while the output from a negative sample is trained to maximize its angle. During inference, the label that produces an output vector closest to the reference vector is selected as the prediction. (**D**) Confusion matrix. The strong diagonal correlation demonstrates the high classification accuracy achieved by the TAS-HD-TSE model. (**E**) Classification accuracy versus training epoch. The TAS-HD-TSE achieves the highest final accuracy of 94.6%, outperforming both the TAS-HD (92.9%) and the traditional approach (90.9%). (**F**) Physical robustness of the TAS. Twenty repeated measurements of a phi-bit’s phase exhibit a maximum deviation of only 1.2° (a relative error of 0.5%). The inset illustrates that this low 0.5% error is critical for achieving smooth learning, in contrast to the degraded performance of a system with 2% error like traditional memristors. (**G**) Power consumption benchmark. The TAS exhibits an ultralow power consumption of 3 × 10^−8^ W, outperforming state-of-the-art FETs (*60*) (2 × 10^−7^ W) and memristors (*61*) (6 × 10^−7^ W).

We implemented a physical local learning algorithm (*58*, *41*) to solve a MNIST handwritten digit recognition task (*59*). As shown in Fig. 4A, the process begins by mapping both the image data (pixel intensity and location) and the class labels (blue for the correct digit and red for an incorrect one) into distinct input frequencies. The TAS processes these frequencies and, through its nonlinear wave dynamics, generates a high-dimensional feature representation composed of multiple phi-bits. These phi-bit sets are then passed to a readout layer, which consists of a fixed random convolutional network followed by a single trainable layer. A contrastive loss is calculated on the basis of the cosine similarity (goodness) of positive versus negative samples, and the update signal is sent back only to the trainable layer.

TAS improves the process in two notable ways. First, TAS enables a unique data categorization strategy, type-specific encoding (TSE), at the image-to-frequencies stage (Fig. 4, A and B). Unlike conventional approaches that process all data uniformly, we map different data types to distinct phi-bit sets. Continuous data, such as pixel intensity and location, are mapped onto one phi-bit set of frequencies (*f*_2_ and *f*_3_), while discrete class labels are mapped to another phi-bit set (*f*_1_ and *f*_4_) (figs. S4 and S5). This leverages the device’s parallel architecture to handle varied information streams simultaneously. Second, the TAS enhances data separability by mapping multiple driving frequencies into a HD phi-bit state that redistributes information and exposes previously obscured features, allowing a small readout layer to train faster and achieve higher accuracy on both the training and validation sets (Fig. 4, A and C). During training, outputs for the correct label rotate toward a reference vector while outputs for all other labels rotate away, so inference reduces to nearest-angle matching (*41*).

The data categorization strategy, combined with the TAS’s HD outputs, produces highly accurate digit classification, as demonstrated by the confusion matrix (Fig. 4D). We benchmarked three models: a traditional model that feeds the data directly into the neural network without HD processing or TSE; a TAS-HD model that uses TAS-generated HD features but no TSE; and a TAS-HD-TSE model, which combines HD features with TSE and follows the scheme shown in Fig. 4 (A to C). Figure 4E shows that the performance of these models demonstrates a hierarchy. The TAS-HD-TSE model achieves the highest final accuracy of 94.6%, outperforming the TAS-HD (92.9%) and the traditional approach (90.9%). The results indicate that both physical HD processing and the data categorization strategy provide distinct, cumulative advantages.

We examine the TAS’s endurance by conducting 20 repeated measurements (Fig. 4F). The results show a maximum phase deviation of only 1.2° and a low relative error of 0.5%, demonstrating the robustness of TAS. This high degree of stability is critical for computation, as learning algorithms rely on a consistent and predictable relationship between a system’s inputs and its outputs. The Fig. 4F inset shows that the low-error (0.5%) system achieves smooth, high-accuracy learning. When the error increases to 2%, a level comparable to traditional memristors, the system suffers from noisy, degraded performance. We benchmark the TAS’s power consumption by estimating the power flux of the acoustic wave inside the waveguides. The system exhibits an ultralow power consumption of 3 × 10^−8^ W, outperforming state-of-the-art electrical devices such as FETs (*60*) and memristors (*61*) (Fig. 4G). Given that power consumption scales quadratically with cross-sectional area, further downscaling of the TAS enables low power consumption down to the sub-femtojoule level.

## DISCUSSION

In addition to intrinsic acoustic energy consumption, the TAS includes an external digital overhead for phi-bit extraction. Specifically, the output is acquired in the time domain and converted to phi-bit phases by fast Fourier transform–based readout, which presently consumes ~1 ms and ~1 μJ per measurement. Each measurement simultaneously provides all phi-bit phases. This overhead can be reduced by tighter hardware integration. For example by implementing the spectral extraction on an FPGA and generating the multitone drive signals with frequency synthesizers (*62*). Further miniaturization can be achieved by implementing silicon-based phononic waveguides (*63*, *64*), with graphene providing controllable mechanical coupling (*65*) and thin-film AlN serving as complementary metal-oxide semiconductor–compatible transducers (*66*).

In conclusion, we have harnessed nonlinear acoustic wave dynamics in coupled waveguides to realize TAS. The intrinsic generation of HD, multivariate phi-bit states in TAS provides massively parallel computational units within a single device. These phi-bits can be manipulated through both analog control and digital-like switching, allowing a single TAS to mimic diverse biorealistic functionalities, including reconfigurable synaptic plasticity (*42*), bilingual synaptic response (*43*), and neuromodulation (*44*). TAS can be easily scaled up to extend computational capacity, emulate multiple neuromodulators, and enable a unique TSE strategy that maps different data types to distinct phi-bit sets. Together, TAS is suitable for HD parallel computing tasks and eliminates the area and energy overhead of conventional devices and interconnects (*7*, *28*, *29*), demonstrating high accuracy, rapid convergence, and robustness at ultralow power. While demonstrated here with a specific acoustic system, the underlying principle of using nonlinear TA waves to physically generate a HD computing space presents a path towards higher-level neuromorphic hardware. Further scaling of the TAS will enable the development of dense, ultralow-power systems that can implement complex, brain-inspired computational models for the next generation of AI.

## MATERIALS AND METHODS

### Experimental setup

The TAS is a nonlinear metamaterial composed of three elastically coupled, finite-length aluminum rods (McMaster-Carr 6061). Each rod has a one-half-inch diameter, a length of 0.609 m, and a density of 2660 kg/m^3^. The rods are arranged in a linear array, with epoxy resin (KwikWeld Syringe) filling the lateral gaps to provide elastic coupling.

Acoustic waves are generated and detected using two sets of ultrasonic longitudinal contact transducers (Olympus IMS, V133-RM) attached to the ends of the rods, using a thin layer of honey as a coupling agent. To drive the system, transducers are actuated by waveform generators (B&K Precision 4055B) connected through high-bandwidth linear amplifiers (PD200). A corresponding set of three detecting transducers is connected to an oscilloscope (Tektronix MDO3024) to measure the output displacement field. The entire array is suspended by thin threads for acoustic isolation, and a central computer controls the experiment and performs data processing by interfacing with the generators and oscilloscope.

### Measurement and data processing

During an experiment, the waveguides are driven with continuous sinusoidal signals at distinct frequencies at room temperature. The temporal signals from the three detecting transducers are captured by an oscilloscope. The captured time-domain signals are then processed using a fast Fourier transform to obtain the frequency spectra of the output channels. These spectra contain peaks at the primary driving frequencies as well as at numerous nonlinear mixing frequencies (*f_p, q_ = pf*_1_ *+ qf*_2_). At each of these peak frequencies, the complex amplitude (magnitude *C_j_* and phase φ*_j_* of the signal from each of the three waveguides is extracted. The state of a logical phi-bit is characterized by the relative phase differences between the waveguides, calculated from the extracted phases (e.g., φ^12^ *=* φ^2^ − φ^1^and φ^13^ *=* φ^3^ − φ^1^).

### Iris classification task

The standard Iris dataset, containing 150 samples across three classes, was used for the classification task. The features of the dataset were scaled to a range of [0.1, 1.0] using a min-max scaler. The data were subsequently split into an 80% training set and a 20% testing set. The computational model uses a hybrid physical-digital architecture, where the TAS functions as a fixed, nontrainable feature expander. The scaled features (petal length, petal width, and sepal length) of each Iris data point were linearly mapped to distinct input frequencies (*f*_1_, *f*_2_, and *f*_3_) within a 3 kHz range, based at 43, 50, and 58 kHz, respectively. The TAS, interfaced via a MATLAB engine, processes these three frequency inputs to produce a 12-dimensional phi-bit–based vector. A trainable digital readout layer, consisting of a single linear layer with 12 input features and three output neurons, was implemented in PyTorch to perform the final classification. The training process was performed in two stages, consistent with a reservoir computing approach. First, the entire training and testing datasets were passed through the physical phi-bit system in efficient batches to generate a static set of 12-dimensional feature vectors. Second, only the digital readout layer was trained on this fixed feature set using a standard backpropagation algorithm. The layer was trained for 100 epochs with a batch size of 16 using the Adam optimizer (learning rate = 0.005) and a cross-entropy loss function. The final classification accuracy was evaluated on the test set.

### MNIST digit recognition task

The standard MNIST dataset of 28 × 28 handwritten digits was used, with all images normalized. To incorporate label information for the learning algorithm, each image was padded to a size of 38 × 28. The integer class label (0 to 9) was then mapped by setting the corresponding row in the top 10 padded rows to the maximum pixel intensity.

Three models were benchmarked, each consisting of a fixed, nontrainable physical front-end followed by a trainable digital layer of 500 neurons. Traditional model: The 38 × 28 input is directly fed into the neural network without TAS processing. TAS-HD model: The physical front-end first applies a phi-bit processing step. The pixel intensity and location of the central 28 × 28 digit are mapped to two distinct frequencies (*f*_2_ and *f*_3_). The simulated physical responses of the TAS to these frequencies, loaded from experimental data, are combined to create a phi-bit–based image. This composite image then passes through the same fixed readout layer. TAS-HD-TSE model: This model uses an enhanced data processing step. In addition to the intensity and location mapping, the class label is also mapped to two further frequencies (*f*_1_ and *f*_4_). Their corresponding phi-bit responses are injected as faint vertical stripes onto the composite image to enrich the feature representation before it enters the convolutional network.

All models were trained for 100 epochs using the physical local learning algorithm with a batch size of 256. This is a contrastive, layer-wise method. For each training sample, “positive” (correct label) and “negative” (incorrect label) inputs are created. Each trainable digital layer is then updated for five internal epochs to maximize the cosine similarity of its positive output to a fixed random vector, while minimizing the similarity of its negative output. During inference, the label that produces the highest cumulative similarity score across all layers is chosen as the prediction.
