# Supplementary material for: Topological acoustic synapse for high-dimensional neuromorphic computing
Source: Sci Adv. 2026 Jun 12;12(24):eaec6633. doi: 10.1126/sciadv.aec6633 (PMC13262634; doi:10.1126/sciadv.aec6633)
Supplement: Supplementary file 1 — Supplementary Text Figs. S1 to S7 Table S1 [file sciadv.aec6633_sm.pdf]

Science Advances
AAAS

Supplementary Materials for

Topological acoustic synapse for high-dimensional neuromorphic computing

Jinli Chen *et al.*

Corresponding author: Xiaodong Yan, xyan@arizona.edu

Sci. Adv. **12**, eaec6633 (2026)
DOI: 10.1126/sciadv.aec6633

This PDF file includes:

Supplementary Text
Figs. S1 to S7
Table S1

Section I. Phi-bits computing

A logical phi-bit is a two-state degree of freedom carried by a nonlinear acoustic mode supported by an externally driven structure of three elastically coupled, finite-length acoustic waveguides. When the waveguides are driven at two distinct frequencies f_1 and f_2, secondary nonlinear modes appear at mixed frequencies:

$$f_{\{p,q\}} = pf_1 + qf_2 \; where \; p, q \in \mathbb{Z},; \; f_{\{p,q\}} > 0$$

Operationally, a phi-bit is the classical analogue of a qubit: it is a two-state degree of freedom of an acoustic wave that can be prepared in a coherent superposition with complex amplitudes.

Single phi-bit representation

Let $O_k(f)$ denote the complex spectral amplitude at the detection end of waveguide, where $k \in \{1, 2, 3\}$. For a phi-bit at frequency $f_{\{p,q\}}$, define the relative phases:

$$\varphi^{ij}(f_{\{p,q\}}) = \; arg \; O_i(f_{\{p,q\}}) - \; arg \; O_j(f_{\{p,q\}}), where \; ij \in \{12,13,23\}$$

The displacement field at the detection ends, referenced to waveguide 1 and normalized in amplitude, can be written as a 2×1 vector:

$$U^j = \left(\hat{c}_2 e^{i\,\varphi_{12}^j}, \; \hat{c}_3 e^{i\,\varphi_{13}^j} \right)^T e^{i\,\omega^j t}, \;\; \omega^j = 2\,\pi\, f_j.$$

For state manipulation, it is convenient to separate the temporal phase and retain only the phase subspace as a normalized complex-amplitude state vector (living in a 2D complex Hilbert space h^j):

$$u^j = \left(\frac{1}{\sqrt{2}}\right)\left(e^{i\,\varphi_{12}^j}, \qquad e^{i\,\varphi_{13}^j} \right)^T$$

A logical phi-bit is a two-level, secondary nonlinear mode labeled by the integer pair {p,q} (its mixed-frequency "good quantum number"), whose logical state is fully specified by the two independent relative phases φ^{12}, φ^{13} measured at the waveguide ends.

For P phi-bits, the composite state is the tensor product

$$W = U^1 \otimes \cdots \otimes U^P, \; dim(H) = 2^P,$$

with basis formed by tensor products of single-phi-bit bases. The accessible Hilbert space scales exponentially as *dim(H) = 2^P*. Correlations among phi-bits arise from nonlinear coupling in the physical system, and states may be separable or non-separable (classically "entangled") superpositions in H.

Tuning parameters. The state u^j is controllable by adjusting driving conditions (frequencies, relative driver phases, and/or amplitudes). Experimentally, varying f_1 or the relative phase of the drivers produces smooth "background" evolution of φ_{12}, φ_{13} punctuated by sharp π phase jumps over narrow parameter ranges.

The measured phi-bit phases can be decomposed into a slow background—well-approximated by linear combinations of the primary-mode phases at f_1 and f_2—and resonant jumps; subtracting the background isolates the intrinsic nonlinear response. Driver-phase tuning offers direct, parallel navigation of multi-phi-bit states in Hilbert space, including reproducible ~180° jumps that occur at mode-dependent parameter.

Single-phi-bit gates. Quantum-like phase and Hadamard operations have been demonstrated on phi-bits; changes that produce simultaneous π jumps in φ_{12} and φ_{13} implement specific unitary rotations in h^j.

Two-phi-bit control. A controlled operation can be realized by tuning a parameter interval that triggers a π jump in one phi-bit's phase while leaving the others nearly unchanged, effecting a CNOT-like permutation of complex amplitudes.

Three-phi-bit gate. A nontrivial three-phi-bit unitary (swapping components with added π phases) has been implemented as a single physical action, without decomposition into smaller gates, and can be mapped to an equivalent quantum circuit of multi-qubit gates.

Fourier-transform operations. By engineering representations whose components transform under simultaneous π jumps, one designs single- and multi-phi-bit operations equivalent to the QFT (or Hadamard for N=1). An explicit N=1 construction is:

$$V_0 = e^{i\,\varphi_2}(\sin\varphi_1\,, \cos\varphi_1)^{T}, \qquad \text{where } \varphi_1 = \frac{1}{2}\varphi_{12}, \varphi_2 = \frac{1}{2}\varphi_{13}$$

Under π jumps, $V_0' = \sigma_y V_0$; choosing a pre-matrix X yields F2 X V0 = X σy V0, implementing the QFT action. This scales to N phi-bits by tensor construction.

These phi-bit operations expand the TAS's computational power, enabling it to function as a versatile platform for both neuromorphic and quantum computing.

Section II. Pulse strategy for TAS

Unlike traditional electronic devices that use voltage, TAS are excited by continuous acoustic waves. These input signals are standard sine waves defined by three parameters: driving amplitude, driving frequency, and initial phase. Only the driving frequency affects the system's phi-bits in a continuous manner. In contrast, the amplitude and phase only produce an effect under specific conditions.

To simulate the voltage pulses found in a biological synapse, a "pulse train" of acoustic waves with varying frequencies is applied to the TAS. In this model: The change in driving frequency (either an increase or decrease) corresponds to the pulse's amplitude. The duration for which a frequency is held corresponds to the pulse's width (Fig.S2).

Section III. Encoding and approximating methods in digit recognition task

The four input frequencies affect the system differently due to TAS's geometry. The inputs f_1 and f_4 are applied at the edges of the coupled waveguides, while f_2 and f_3 are applied to the internal waveguides. This physical asymmetry causes the distinct phase responses observed in the φ^{12}, φ^{13}, and φ^{14}, sets as follows (Fig. S4).

The distinct response of the system to each driving frequency allows them to serve different functions and encode different types of information. For the handwritten digit recognition task, we implement the frequency encoding scheme illustrated in Fig. S5.

To simulate the system's behavior, we use a linear approximation method:

$$\Delta\varphi^{\mathrm{ij}}_{\mathrm{af1+bf2}} = a\,\Delta\varphi^{\mathrm{ij}}_{\mathrm{f1}} + b\Delta\varphi^{\mathrm{ij}}_{\mathrm{f2}}.$$

This assumption properly estimates the phi-bit component value unless the π- jump occurs.

The number of useful phi-bits generated from a single drive frequency is set by the number of phase-difference channels that can be read out and by how many of those channels provide non-redundant information in the operating regime. Using the notation $\phi^{ij}_{f_k}$, where i, j label the waveguide pair and f_k is the drive applied to waveguide k, the four-waveguide system provides three phase-difference channels per drive frequency, $\phi^{12}_{f_k}$, $\phi^{13}_{f_k}$, and $\phi^{14}_{f_k}$, giving 12 basic linear phi-bit phases across $k = 1 \dots 4$. Nonlinear mixing generates additional phases at combination frequencies such as $\phi^{ij}_{f_k+f_\ell}$, and for a given relative index the mixing phases follow the approximate linear relation $\phi^{ij}_{pf_k+qf_\ell} \approx p\,\phi^{ij}_{f_k} + q\,\phi^{ij}_{f_\ell}$, so the independent information content is primarily set by the fundamental phases at the driven frequencies, with mixing terms contributing features only when they are decorrelated from the basic channels via π- jump.

Fig. S4 shows that several basic channels are correlated due to device topology: when sweeping $f_1$and f_4, the corresponding phase responses $\phi^{12}_{f_1}$, $\phi^{13}_{f_1}$, $\phi^{14}_{f_1}$and $\phi^{12}_{f_4}$, $\phi^{13}_{f_4}$, $\phi^{14}_{f_4}$exhibit similar trends, consistent with waveguides 1 and 4 occupying edge positions and coupling to the rest of the network through similar paths. When sweeping $f_2$and f_3, the phase responses vary more distinctly, consistent with waveguides 2 and 3 occupying interior positions and experiencing different coupling environments. Accounting for these correlations in the measured operating regime reduces the effective dimensionality of the 12 basic linear phi-bit phases, and the present four-waveguide prototype provides eight phi-bit features that behave effectively independently and are useful for learning.

The TAS supports programmability, and its experimentally demonstrated reconfigurability provides a physical basis for online training by updating the drive frequencies during operation.

Programmability is shown by the ability to continuously tune and target specific phi-bit phases through changes in the drive frequencies. The manuscript (Figs. 1 and 3) demonstrates both coarse tuning and fine

tuning of phi-bit phases, which allows the system to move quickly to a new operating region and then refine the response to reach a desired phase. In addition, the TAS demonstrates programmable synaptic plasticity, including reconfigurable LTP/LTD and switchable excitatory/inhibitory responses, by shaping how phi-bit phases evolve under controlled frequency modulation. These bio-mimicking behaviors allows phi-bit to represent various learning rates and enable more precise recognition.

The TAS can support online training by leveraging this programmability. For example, in the MNIST task, coarse frequency updates can rapidly shift the phi-bits to a new operating region (corresponding to a different digit-class condition), while fine adjustments can further optimize the phi-bit separability for improved classification. In detail, the TAS can first be trained for a given digit class (e.g., “1”) using continuous, analog frequency tuning to gradually refine the phi-bit phases and maximize class separability. Once this training converges, the system can then apply a digital jump in the driving-frequency set to move to a new operating region and rapidly switch the training target to the next digit class (e.g., “2,” then “3,” etc.), without requiring another round of analog fine-tuning for each class.

Section IV. Power Benchmarks for TAS devices

Below, we show how we estimate the energy and power required to pass one spike through a phi-bit device in Fig. 4g. In Phi-bit devices, the 1s pulses are applied during training and synaptic operation. Under this measurement condition and during each pulse period, the energy consumption are composed of the oscillation energy cost during this time.

For any acoustic field, the instantaneous power flux (intensity) I (t) is

$$I(t)= p(t)u(t),$$

where p is acoustic pressure and u is particle velocity.

For a lossless plane progressive wave in a medium with density ρ and sound speed c,

$$u(t) = \frac{p(t)}{\rho c}$$

So, the time–average intensity over one cycle is

$$\langle I(t)\rangle = \langle \frac{p(t)}{\rho c}\rangle = \frac{\Delta p^2}{2\rho c}$$

Where Δp is the radiated pressure amplitude

Power through an aperture of effective area A is

$$P= \langle I\rangle A = \frac{\Delta p^2 A}{2\rho c}$$

The experiment device using Olympus V133, the radiated pressure amplitude is estimated by the acoustic impedance (ρc) of the medium and the particle velocity in the medium u via

$$\Delta p \approx \eta\rho c u,$$

where η~0.1 for a heavily backed NDT probe driven far off resonance. And u=ω d33 V is particle-velocity amplitude. where $d_{33} \approx 200$ pm/V. ω is 2πf and V is driving voltage. For our aluminum rod system, ρ ≈2700 kg/m3 c ≈6320 m/s, the area A= 1cm^2

Plugging *f*=10 kHz, V=5 V, d_{33}≈200 pm/V.

We have

$$P \approx 3\times10^{-8} W$$

Section V. Computation Time for TAS devices

The computation time of the TAS system is determined by three steps in the TAS workflow, and each step is governed by a distinct physical or measurement constraint. In step (i), we convert image information into the same number of TAS driving frequencies. This step is implemented as a straightforward operation, with negligible overhead compared with physical actuation and readout (~1 μs and ~10 nJ per input). In step (ii), acoustic waves are injected into the TAS, and the device responds through transport and settling in the coupled waveguides. The intrinsic response time of this step is mainly set by the acoustic velocity and the effective propagation length through the waveguides and couplers. In the current aluminum-rod prototype, the longitudinal acoustic speed is on the order of kilometers per second and is lower in the epoxy coupling region, so the time-of-flight across the present geometry is sub-millisecond, typically ~10-100 μs (with power consumption on the order of ~10^{-8} W). Reducing the device length decreases this time approximately proportionally. In step (iii), we measure the output waveform in the time domain and extract phi-bit phases in the frequency domain using FFT-based readout. The time for this step is primarily determined by the sampling window required to resolve closely spaced frequency components with sufficient signal-to-noise ratio (i.e., the required frequency resolution and SNR), followed by spectral extraction. In the current setup, this typically corresponds to recording windows on the order of ~1 ms and presently consumes ~1 μJ per measurement to obtain phi-bit phases with high signal-to-noise ratio.

The inference time in the current TAS system is dominated by signal acquisition and spectral readout. Because TAS outputs are encoded in the frequency domain (mixing products at specific combination frequencies), the phi-bit values are obtained by recording a time-domain waveform with sufficient duration to achieve the required frequency resolution and signal-to-noise ratio, followed by FFT-based spectral analysis. The frequency resolution scales inversely with the acquisition time window ($\Delta f \approx 1/T$), and additional averaging may be applied to suppress noise and improve stability. These requirements set the current effective measurement time to approximately the millisecond level per stimulus, depending on the sampling length, averaging strategy, and the bandwidth/noise performance of the electronics.

Increasing the acoustic phase velocity and/or reducing the propagation length directly shortens the intrinsic physical response time by reducing the time-of-flight. Increasing the driving frequency can further enable higher-rate operation and may reduce the acquisition time needed to reach a target signal-to-noise ratio for a fixed detector bandwidth. The most direct pathway to faster operation is a combination of device miniaturization (shorter acoustic paths), higher-frequency integrated transduction, and optimized readout electronics that reduce the time required to extract phi-bit amplitudes with adequate precision.

Section VI. Operation parameters and effect of driving amplitude

This section describes how key structural and operational parameters govern the TAS response, including waveguide dimensions, spacing, and the acoustic intensity that sets the nonlinear operating point.

The waveguide dimensions, including length and cross-sectional area, control TA-wave dynamics by defining the allowed eigenmodes and the spatial field distribution. The waveguide length, 0.609 m in the current prototype, sets the resonance spectrum and the node–antinode pattern that localizes TA energy along the rods. The length also sets the TA time-of-flight, which bounds the intrinsic response speed. The cross-sectional area sets the mechanical impedance and the TA-wave energy density, which tunes the effective nonlinearity, the strength of wave mixing, and the power consumption. In the current TAS, aluminum rods serve as the waveguides and epoxy serves as the compliant coupler. The elastic stiffness and mass density determine the TA-wave dispersion, including the allowed mode frequencies and their dependence on wave number, and therefore the phase and group velocities that transport TA energy through the device.

Waveguide spacing controls the strength of inter-waveguide coupling and the efficiency of TA-wave energy and phase exchange across the network. In the current prototype, adjacent aluminum waveguides are spaced by about 2–3 cm and coupled through an epoxy layer, which corresponds to a moderate-coupling regime. Smaller spacing or stronger coupling increases TA-mode hybridization and enhances nonlinear intermodulation, producing stronger and more correlated phi-bit mixing, whereas larger spacing weakens hybridization and improves channel isolation. In the weak-coupling limit, phi-bits behave more independently, but a stronger drive is typically required to reach the same output amplitude and maintain stable phi-bit outputs, which increases power consumption.

The acoustic wave intensity in each waveguide sets the nonlinear operating point of the TA-wave response. Larger acoustic amplitudes enhance nonlinear wave mixing and improve phi-bit signal-to-noise, with higher energy consumption. An amplitude-sweep measurement was performed with all parameters fixed except the driving amplitude applied to Rod 1. The Rod-1 drive amplitude was swept from 10 to 20 V while the driving frequencies and other conditions were held constant, and the resulting phi-bit phase evolution is shown in Fig. S6. A clear threshold occurs at approximately 11 V, where the phi-bit phases change abruptly, indicating a transition between TAS operating regimes.

Figures

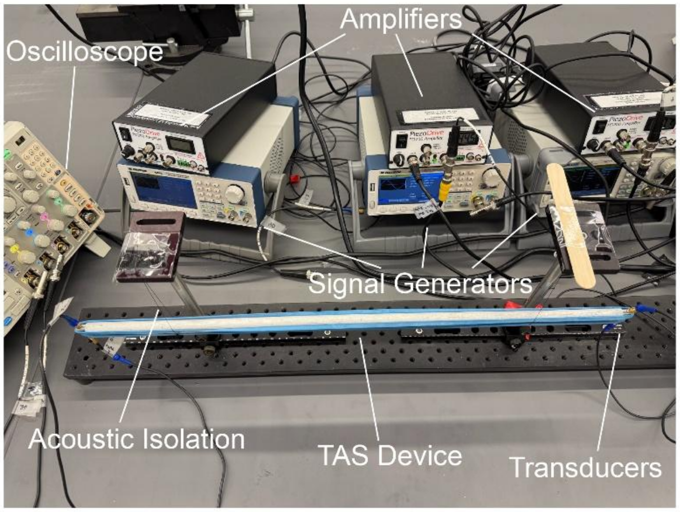

Fig. S1. Device Picture. Acoustic waves are generated and detected using two sets of ultrasonic longitudinal contact transducers attached to the ends of the rods, using a thin layer of honey as a coupling agent. To drive the system, transducers are actuated by waveform generators connected through high-bandwidth linear amplifiers. A corresponding set of three detecting transducers is connected to an oscilloscope to measure the output displacement field. The entire array is suspended by thin threads for acoustic isolation, and a central computer controls the experiment and performs data processing by interfacing with the generators and oscilloscope.

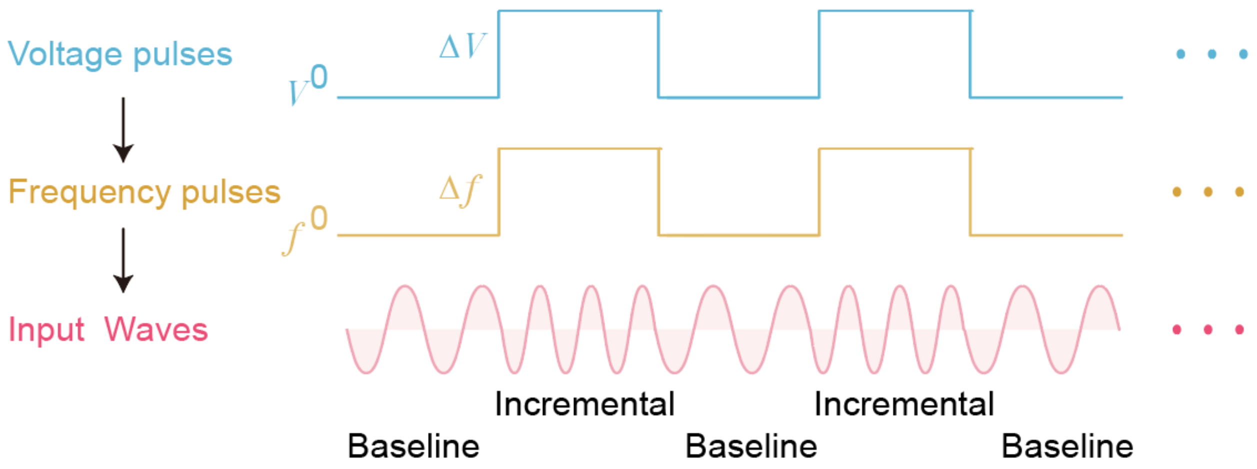

Fig. S2. TAS pulse strategy. This figure illustrates how TAS translates a traditional synaptic signal into an acoustic one. The top panel shows a standard voltage pulse train, which is used as a model for the frequency pulse train shown in the middle plot. A baseline acoustic frequency (f_0) is temporarily increased by an amount (Δf) to create an analogous pulse. The bottom plot shows the resulting input acoustic wave, where the Baseline periods have a lower frequency and the Incremental periods show a higher frequency.

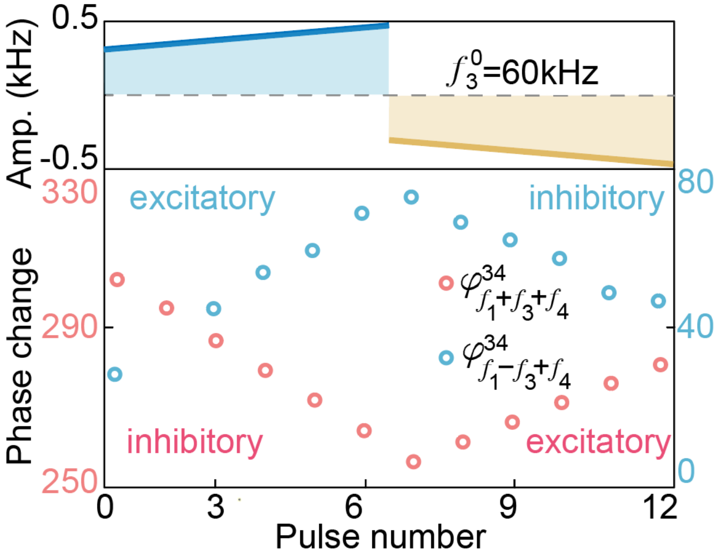

Fig. S3. Phase change versus pulse number in 4-waveguide system. The input pulse amplitude is modulated over time (top panel). The modulation switches the learning behavior between inhibitory and excitatory responses.

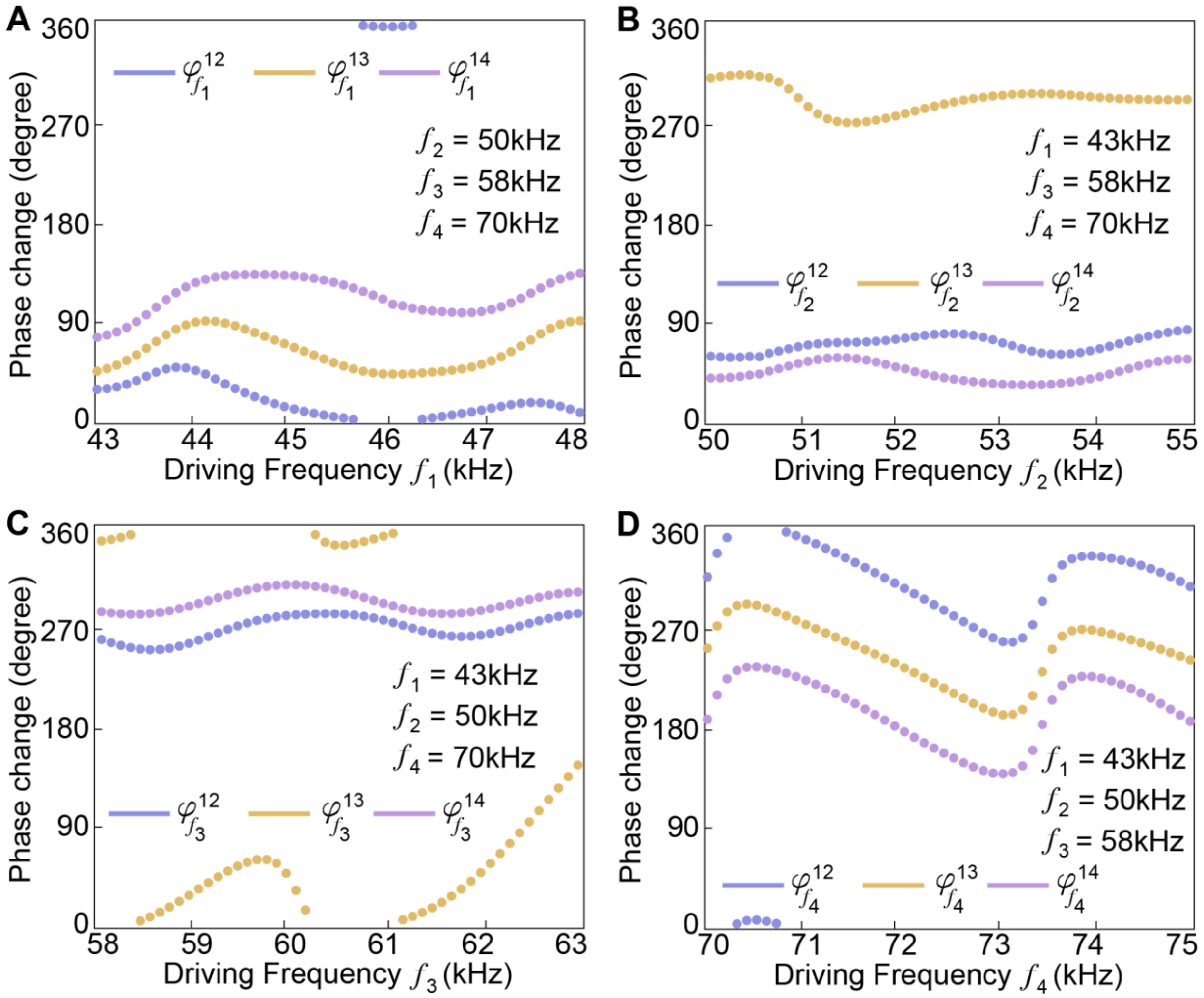

Fig. S4. Different components of same phi-bit vary with driving frequencies. The TAS exhibits distinct phase responses due to geometric asymmetry. Phase components of a single phi-bit were measured as a function of each of the four input driving frequencies (f_1, f_2, f_3, f_4). (**A**, **B**, **C**, **D**): Evolution of the φ^{12}(blue), φ^{13} (brown), and φ^{14} (purple) phase components as each respective input frequency is swept while the other three are held constant. The inputs f_1 and f_4 are applied to the outer waveguides, while f_2 and f_3 are applied to the inner waveguides, showing different coupling behaviors.

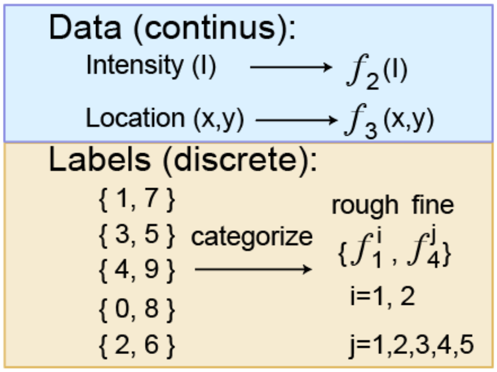

Fig. S5. Type specific encoding scheme of HD phi-bit approach in the digit recognition task. The strategy maps different types of information to specific driving frequencies. Continuous data, such as pixel intensity (I) and location (x,y), are encoded onto frequencies f_2 and f_3, respectively. Discrete class labels are categorized into groups (e.g., rough, fine) and mapped onto the remaining frequencies, f_1 and f_4.

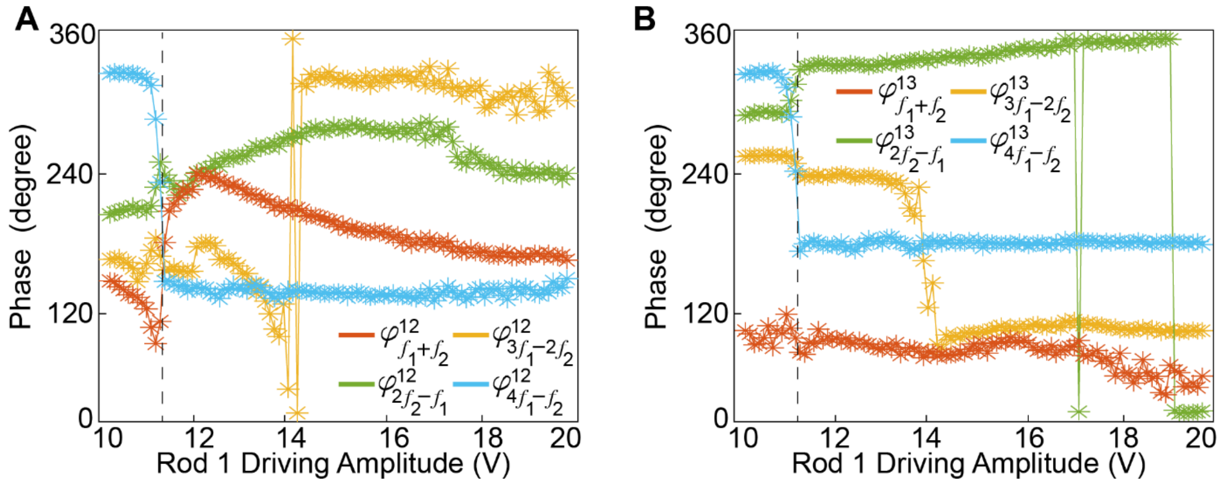

Fig. S6. Effect of driving amplitude on phi-bit phases. A three-waveguide TAS is driven at fixed frequencies $f_1 = 50$ kHz and $f_2 = 54$ kHz. The Rod-1 driving amplitude $A_1$is swept from 10 to 20 V while monitoring the phi-bits at $f_1 + f_2$, $2f_2 - f_1$, $3f_1 - 2f_2$, and $4f_1 - f_2$. (**A**) Measured phases of ϕ^{12}. (**B**) Measured phases of ϕ^{13}. A distinct amplitude threshold near $\sim$ 11 V (dashed line) produces abrupt phase changes across multiple phi-bits, showing a transition between two TAS operating regimes.

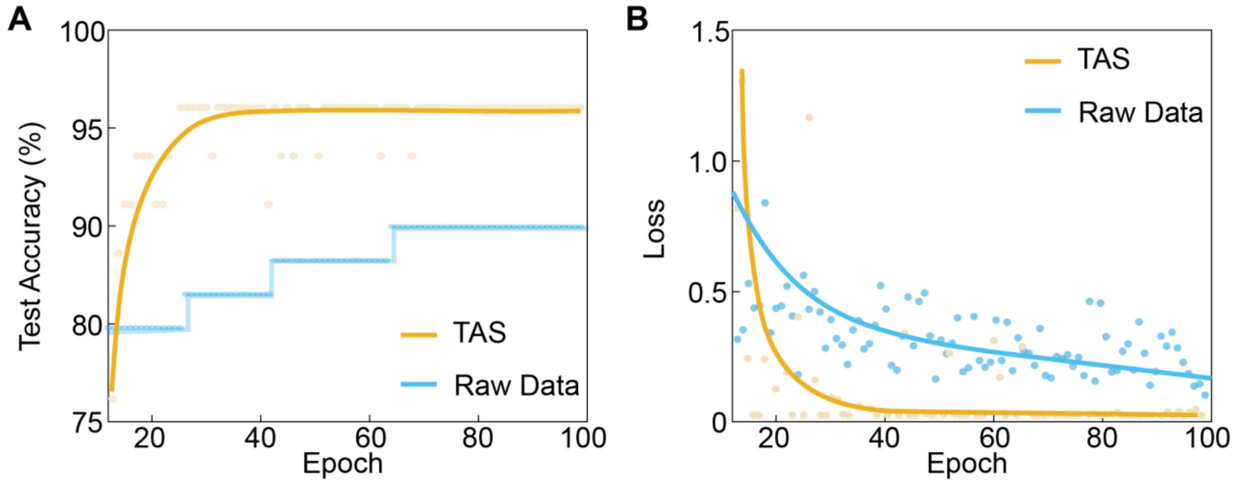

Fig. S7. Convergence comparison with minimal preprocessing. (**A**) Test accuracy and (**B**) training loss versus epoch for a single linear readout classifier trained on two different inputs: TAS features (yellow) and raw data (blue). For the TAS case, the Iris features are mapped to driving frequencies and the TAS generates a 12-dimensional phi-bit phase feature vector that is fed to the same linear readout layer. For the raw-data baseline, the same Iris features are fed directly into the same readout layer without TAS processing. TAS-based neural network converges rapidly to ~96% accuracy with low loss and improved stability, while the raw-data baseline converges more slowly and saturates at a lower terminal accuracy with larger fluctuations.

Tables

Table S1. Summary of TAS roles, trainable parameters, and training objectives in tasks

Task	Role of TAS	Trainable Parameters	Training Goal
Iris Classification	Physical Reservoir: TAS acts as a fixed, non-trainable feature expander.	Digital Readout Layer: A single linear layer with three neurons.	Minimize cross-entropy loss using backpropagation.
MNIST Recognition	Physical processor: TAS generates HD phi-bit representations of image-label pairs.	TAS-based Output Layer Weights: A single layer of digital neurons appends to TAS.	Maximize cosine similarity (goodness) of the TAS response to a reference vector for positive samples.
